# Selective Effects of Acutely Administered N‐Acetyl‐Cysteine in Rodent Models of Nicotine‐Conditioned Behaviours

**DOI:** 10.1111/adb.70051

**Published:** 2025-08-29

**Authors:** Kelsey Stoddart, Michael Davies, Jamie Oughton, Emma Malcolm, Shakir D. AlSharari, Mohammed Shoaib

**Affiliations:** ^1^ Institute of Neuroscience Newcastle University Newcastle UK; ^2^ Department of Pharmacology and Toxicology, College of Pharmacy King Saud University Riyadh Saudi Arabia; ^3^ College Park Hatfield England

**Keywords:** context‐dependent locomotor sensitisation, discrimination, drug, glutamate, N‐acetylcysteine, nicotine dependence

## Abstract

Chronic nicotine administration leads to neuroadaptations, an important process in nicotine and tobacco dependence for which treatments are limited. The cysteine pro‐drug, N‐acetyl‐cysteine (NAC), is a promising glutamatergic agent that has shown some clinical efficacy in reducing nicotine use in humans. The purpose of this study was to examine NAC in two rodent models of nicotine dependence. NAC (0, 5, 20, 50 and 100 mg/kg) was examined on locomotor activity in groups of rats previously exposed to nicotine or saline. In the second experiment, NAC (0, 50 and 100 mg/kg i.p.) was evaluated against the discriminative stimulus effects of nicotine (0.2 mg/kg) using a two‐lever procedure under a tandem schedule (VI10”‐FR10) of food reinforcement. Pre‐treatment with NAC in doses greater than 20 mg/kg attenuated the expression of conditioned hyperactivity when rats were placed in locomotor boxes previously paired with chronic nicotine administration. The same doses of NAC had modest effects in attenuating nicotine‐stimulated hyperactivity in nicotine‐treated or saline‐treated rats tested in the same locomotor boxes. In the discrimination task, NAC did not generalise to the nicotine stimulus and nor did it modify the dose–response curve to nicotine, suggesting that NAC may not modify the subjective effects of nicotine. These results suggest NAC selectively attenuates conditioned responses to nicotine‐paired stimuli without modifying nicotine‐induced hyperactivity or the discriminative stimulus effects of nicotine. Thus, the study proposes that if NAC was to act in a similar selective manner in humans, the specific action of NAC to attenuate conditioned responses may limit its potential as a treatment to manage nicotine dependence.

## 1 Introduction

Smoking‐related illnesses are responsible for over 10% of deaths worldwide and are the leading cause of preventable mortality in high‐income countries [1, 2]. Despite the many therapies available to aid smoking cessation [3], relapse rates remain at over 50% within the first 12 months of quitting [4]. Therefore, more effective therapies are urgently needed to promote long periods of abstinence and help reduce the prevalence of smoking‐related mortalities.

It is widely accepted that nicotine is the primary addictive agent in tobacco smoke and its reinforcing properties have been extensively demonstrated pre‐clinically [5, 6]. The positive reinforcing effects of nicotine, including mild euphoria, anxiolytic effects, analgesic effects, and suppressed appetite, promote the continued practice of tobacco smoking [7, 8]. Conditioned effects of smoking and unpleasant effects of withdrawal are equally important in the maintenance of smoking behaviour, and together, they have also been shown to contribute to the development of nicotine dependence [9, 10].

Current therapies for tobacco smoking cessation include a combination of pharmacological treatments, such as nicotine replacement therapy (NRT), varenicline, and bupropion, along with behavioural interventions like counselling and support groups [11]. Despite these options, a systematic review published in 2024 highlighted the limited efficacy of these interventions [12].

Chronic exposure to nicotine leads to neuroadaptations in dopamine, GABA and glutamate neurotransmitter systems, all of which are modulators of synaptic plasticity [13], along with accumulation of neurotrophins that regulate the morphological structure of the neurons [14, 15]. Specifically, it is thought that adaptations in glutamatergic neurotransmission play a significant role in the conditioned effects of chronic drug taking [16]. Levels of glutamate in the nucleus accumbens have been shown to increase following the presentation of a stimulus previously paired with drugs like cocaine [17]. In addition, glutamatergic systems are believed to be largely involved in assisting relapse following drug‐seeking behaviour [18]; therefore, the regulation of this system serves as a potential target for smoking relapse prevention.

N‐acetyl‐cysteine (NAC), a prodrug of the endogenous amino acid L‐cysteine, has been used in clinical practice for over 30 years [19]. It has many indications for use, including paracetamol overdose [20], chronic obstructive pulmonary disease (COPD) [21], and contrast‐induced nephropathy, with a recent review suggesting that it may be beneficial in the treatment of psychiatric disorders [22]. NAC is rapidly absorbed after entering the body, quickly deacetylated and further oxidised to cysteine [23]. Cysteine commonly enters the cell via the cystine/glutamate antiporter (system x_c_^−^), where it can be reduced back to cysteine as the rate limiting step in the production of the antioxidant glutathione (GSH) [24]. In addition to increasing levels of GSH, NAC activates the system x_c_^−^ causing the release of extracellular glutamate; an action central to its therapeutic efficacy in the CNS [24, 25].

Extracellular glutamate is essential for the regulation of the glutamatergic system, principally via metabotropic glutamate receptors; mGluR2/3 and mGluR5 [26, 27]. Following chronic cocaine or self‐administered nicotine administration, levels of mGluR2/3 are reduced in the nucleus accumbens and VTA [28, 29]. The resulting effect is decreased ability to regulate synaptic glutamate release and neurotransmission [29]. Animals undergoing extinction from chronic drug administration show a downregulation of mGluR5 expression in the NAc [30], which when coupled with decreased mGluR2/3, is believed to alter long‐term depression and long‐term potentiation in glutamate signalling [31]. The combined effect of these drug‐induced alterations is an overall dysregulation in glutamate neurotransmission; a neuroadaptation thought to be largely involved in nicotine dependence [32]. Specifically, it has been proposed that up‐regulated GluN2A, GluN2B receptor subunits, and rapid synaptic potentiation in the accumbens contribute to cue‐induced relapse to nicotine use [33].

NAC restores glutamate dyshomeostasis induced by chronic nicotine use by modulating the glutamatergic system through its action on glutamate transporters and receptors. Chronic nicotine use leads to an imbalance in glutamate levels, which contributes to addiction and withdrawal symptoms [34].

Several randomised controlled trials have explored the efficacy of NAC in reducing cravings and withdrawal symptoms in individuals with substance use disorders (SUDs). However, the results have been mixed [35]. Some studies have shown that NAC can significantly reduce cravings for substances such as alcohol, cocaine, and nicotine [34, 36, 37, 38]). For example, a meta‐analysis of 11 RCTs found that NAC reduced craving ratings compared to placebo, but the evidence was considered weak, and there were no significant differences in withdrawal symptoms or side effects [39, 40]). Other studies have reported no significant difference between NAC and placebo in reducing cravings. The high heterogeneity and potential publication bias in these trials suggest that more research is needed to confirm the efficacy of NAC in treating SUDs [35].

NAC has been shown to attenuate cue‐induced reinstatement of nicotine‐seeking behaviour in rodents, at doses ≥ 60 mg/kg [41, 42, 43, 44, 45]. To evaluate the selectivity of NAC, its effects on cue‐induced reinstatement of food‐seeking have been measured in parallel to reinstatement of nicotine‐seeking behaviour. Doses of NAC greater than 60 m/kg were observed to exert a non‐specific as it reduced cue‐induced reinstatement of food‐seeking behaviour [42, 45]. In contrast to this finding, Bowers et al. [46] demonstrated that NAC doses below 60 mg/kg had no effect on operant responding maintained by food reinforcement [46], suggesting that the behavioural selectivity of NAC remains unclear. NAC significantly decreases nicotine self‐administration under a fixed ratio schedule of reinforcement [45], as well as reducing the development of nicotine conditioned rewarding effects [46], suggesting that the rewarding properties of nicotine are impacted by NAC. In addition, the physical symptoms associated with nicotine withdrawal have been shown to decrease following NAC administration [46].

The present study aims to expand the current understanding of NACs effects in preclinical models of nicotine dependence. Specifically, the experiments evaluated the impact of NAC administration on the conditioned, pharmacological and subjective effects of nicotine, using two well‐established rodent models of nicotine dependence; conditioned hyperactivity in a context‐dependent locomotor sensitisation paradigm and a conventional two‐lever drug discrimination task.

It is known that chronic nicotine administration results in sensitisation to the stimulant effects, i.e., enhanced locomotor activity [47, 48]. In part, this is due to nicotine's direct pharmacological action and upregulation of nicotinic receptors [49]; however, the environmental context of nicotine administration is also known to facilitate the development of behavioural sensitisation [50]. This study utilises a nicotine sensitisation model to establish the conditions under which conditioned hyperactivity can be expressed. The first experiment will examine acute doses of NAC against the conditioned hyperactivity responses and will be compared against responses to nicotine‐induced hyperactivity. Considering NAC's ability to attenuate cue‐induced reinstatement of nicotine‐seeking behaviours [41, 42, 43, 44, 45], it is hypothesised that NAC will attenuate the conditioned hyperactivity responses in environments previously associated with nicotine administration.

Drug discrimination is a well‐established model for capturing the subjective effects associated with the discriminative stimulus properties of nicotine [51, 52]. Despite a clinical study demonstrating NAC ability to attenuate the number of cigarettes smoked [28], there is a lack of preclinical research evaluating the impact of NAC administration on the subjective effects of nicotine.

## 2 Methods and Materials

### 2.1 Animals

Male hooded Lister rats (Envigo, Bicester, United Kingdom) were housed in a temperature (20–22 °C) and humidity‐controlled room on a regular 12‐h light/dark cycle (0730 to 1930 h). Upon arrival, they were habituated to the new environment for at least 7 days before any experimental procedures commenced. Rats were 8 weeks old and weighed between 220 and 250 g at the start of the experiment. All procedures complied with local and national ethical requirements and were carried out according to the Animals (Scientific Procedures) Act 1986, in addition to the EU Directive 2010/63/EU on the protection of Animals for Scientific Purposes, under a project licence issued by the UK Home Office.

### 2.2 Conditioned Hyperactivity Locomotor Sensitisation Experiment

Locomotor activity boxes (40 cm × 40 cm × 40 cm) (Opto‐Varimex, Columbus Instruments, Colorado) with photocell beams evenly spaced along the X and Y planes were used to measure locomotor activity. Beam interruptions were processed on a personal computer using Auto‐Track System software (Columbus Instruments, CO).

#### 2.2.1 Experimental Design

**FIGURE 1:**
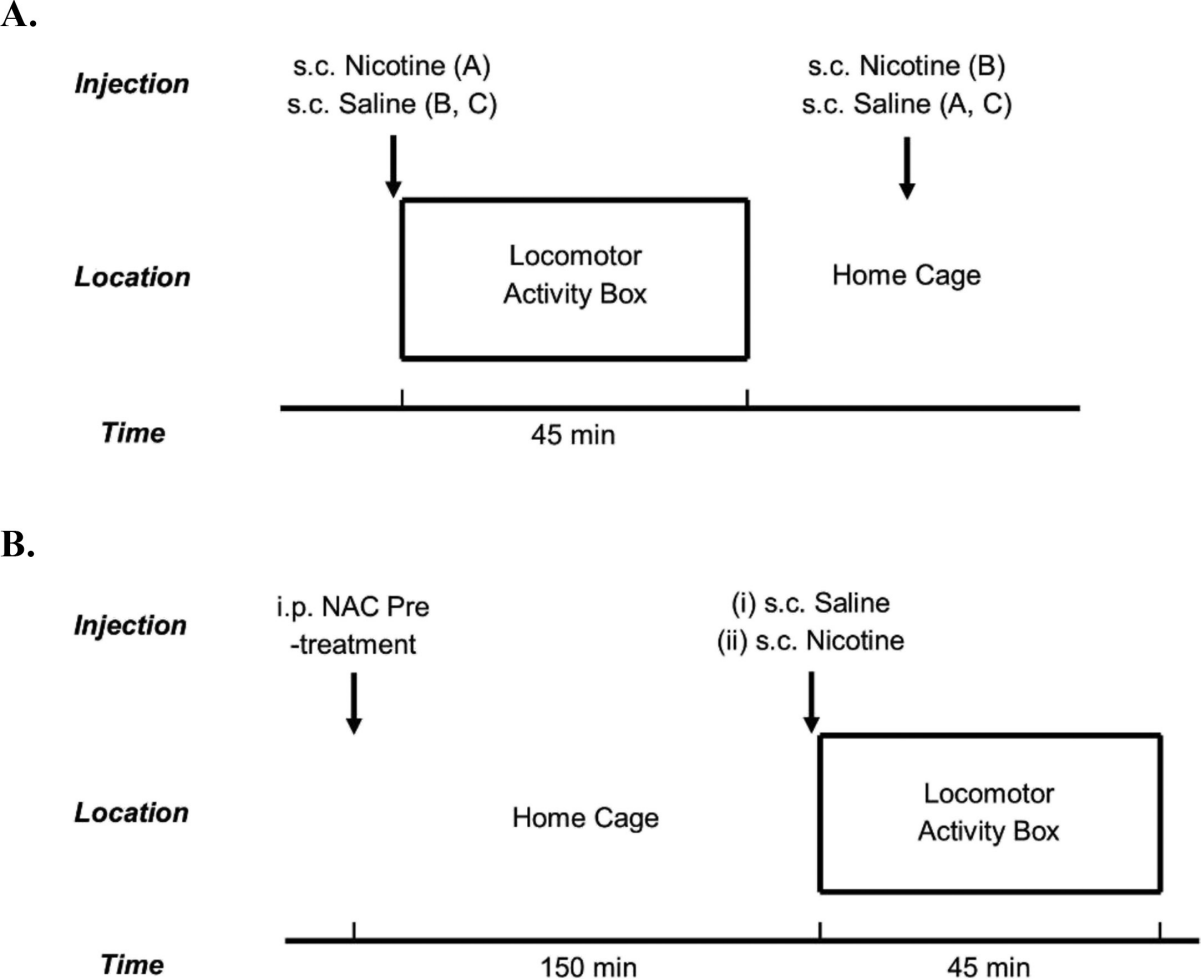
Context‐dependent locomotor sensitisation experimental design. (A) *Training phase*: Immediately before being placed in the locomotor activity box, rats received 0.4 mg/kg SC nicotine or saline injection SC (group dependent). Locomotor activity was then measured for 45 min, and the rats were subsequently returned to their cages. Here, they received 0.4 mg/kg SC nicotine or saline injection SC. Groups: NIC‐paired (A) (*n* = 7), NIC‐unpaired (B) (*n* = 6), Control (C) (*n* = 6). (B) *Test phase*: Animals received NAC pre‐treatment 150 min prior to the start of the test session. Animals were then injected with either (i) saline SC or (ii) 0.4 mg/kg SC nicotine immediately prior to the test session, where locomotor activity was measured for 45 min. All NAC pre‐treatment doses were tested with both saline administration and nicotine administration.

Nineteen male hooded Lister rats were randomly assigned to one of three groups: nicotine‐paired (NIC‐paired; 0.4 mg/kg s.c.), nicotine‐unpaired (NIC‐unpaired; 0.4 mg/kg s.c.) and Control. Figure 1 shows the experimental design for the *conditioning phase* (Figure 1A) and the *test phase* (Figure 1B). Conditioning sessions were carried out once daily for 14 days, over consecutive days. A randomised order of NAC doses (0, 5, 20, 50, 100 mg/kg, IP) were tested as a pre‐treatment (150 min) during the test phase. Tests were conducted twice weekly with the same treatments administered as in the conditioning days during these intervening days.

### 2.3 Drug Discrimination Experiment

Twelve hooded Lister rats were housed in groups of four with water available ad libitum. Rats were maintained at 85% of their free feeding weight by restricting food to 16 g/day for each rat. Discrimination training and test sessions occurred twice a day at 0900 and 1500 hours. Operant conditioning sessions, in which lever pressing resulted in food pellets (45 mg), took place in standard operant conditioning chambers equipped with retractable levers positioned on either side of a food receptacle (Campden Instruments Ltd, Loughborough, United Kingdom).

#### 2.3.1 Training Phase

Operant conditioning sessions were 30 min in duration. No levers were present in the operant conditioning chambers, and food pellets were delivered under a variable interval (VI) schedule of 30 s. This was followed by sessions in which one lever was introduced into the chamber, and rats were trained for 3 days to produce an operant response to obtain food pellets on a continuous reinforcement schedule (i.e., one lever press was required to receive one food pellet). A fixed ratio (FR) schedule was introduced which was progressively increased to FR10 (i.e., required 10 consecutive lever presses to obtain one food pellet). Once the rats responded consistently on the FR10 schedule, the original lever was removed, and another lever introduced at the opposite end of the chamber. The training process was repeated for this lever.

Operant conditioning sessions were reduced to 15 min in duration for nicotine discrimination training. Rats were trained to discriminate nicotine (0.2 mg/kg, SC) from saline, administered 10 min before the operant conditioning sessions, under a tandem VI10” FR10 schedule of food reinforcement. In half of the animals, operant responses on the right‐hand lever were reinforced by food presentation following nicotine injection, and the responses on the left‐hand lever were reinforced by food following saline injection. In the other half of the animals, the allocation of drug‐appropriate lever was reversed. The order of nicotine and saline training sessions that lasted 15 min were randomised, except for the requirement that no more than three successive injections of the same condition were ever administered. A stable level of discrimination performance was required before testing with NAC commenced (i.e., 8 consecutive ‘correct’ lever selections in the last 10 sessions).

#### 2.3.2 Test Phase

The effect of NAC (0, 50 and 100 mg/kg) on the discrimination of a range of nicotine doses (0, 0.025, 0.05, 0.1, 0.2 and 0.4 mg/kg) was tested, resulting in 18 within‐subject tests conducted in a randomised order. Rats were pre‐treated with NAC or vehicle 150 min before the test session and then administered nicotine or saline 10 min before the test session. The test sessions performed under extinction were 5 min in length, where rats had access to both levers and responses did not result in food presentation. Two test sessions were conducted each week, and training sessions resumed during the intervening days to prevent extinction of the discrimination learning.

### 2.4 Drugs

All drugs were dissolved in 0.9% physiological saline to provide the necessary concentrations. The pH of the nicotine hydrogen tartrate salt (Sigma‐Aldrich Co. Ltd, Gillingham, United Kingdom) was adjusted to 7.0 ± 0.2 using diluted sodium hydroxide. Nicotine was administered subcutaneously (s.c.) with a 10 min presession interval. Doses of NAC (Sigma‐Aldrich Co. Ltd, Gillingham, United Kingdom), administered intraperitoneally (i.p.) were selected based on published literature with a pre‐session interval of 150 min. Doses of both drugs are expressed as the base.

### 2.5 Statistical Analysis

Analyses were conducted in IBM SPSS Statistics Version 24. Statistical significance was set at *p* < 0.05 and LSD post hoc tests (pairwise comparisons) are reported.

#### 2.5.1 Conditioned Locomotor Hyperactivity

A three‐way analyses of variance (ANOVAs) for repeated measures was used to determine the effect of conditioned group (NIC‐paired, NIC‐unpaired and Control), NAC pre‐treatment and nicotine administration. Once significant interactions were identified, subsequent two‐way and one‐way ANOVAs were used to determine the effects of the key factors to establish the effects of NAC pre‐treatment within specific treatment groups.

#### 2.5.2 Drug Discrimination

A two‐way ANOVA for repeated measures was used to investigate interactions between the two factors: NAC pre‐treatment and nicotine dose, on the percentage of nicotine‐appropriate lever‐press responses. The effects of NAC pre‐treatment and nicotine dose on total lever responses were examined using a two‐way ANOVA for repeated measures. In cases where response rates were supressed to less than a total of 10 responses, the percentage measure of nicotine‐appropriate responses was not utilised for the statistical analysis.

## 3 Results

### 3.1 Conditioned Hyperactivity

#### 3.1.1 Total Activity Over 45 min

**FIGURE 2:**
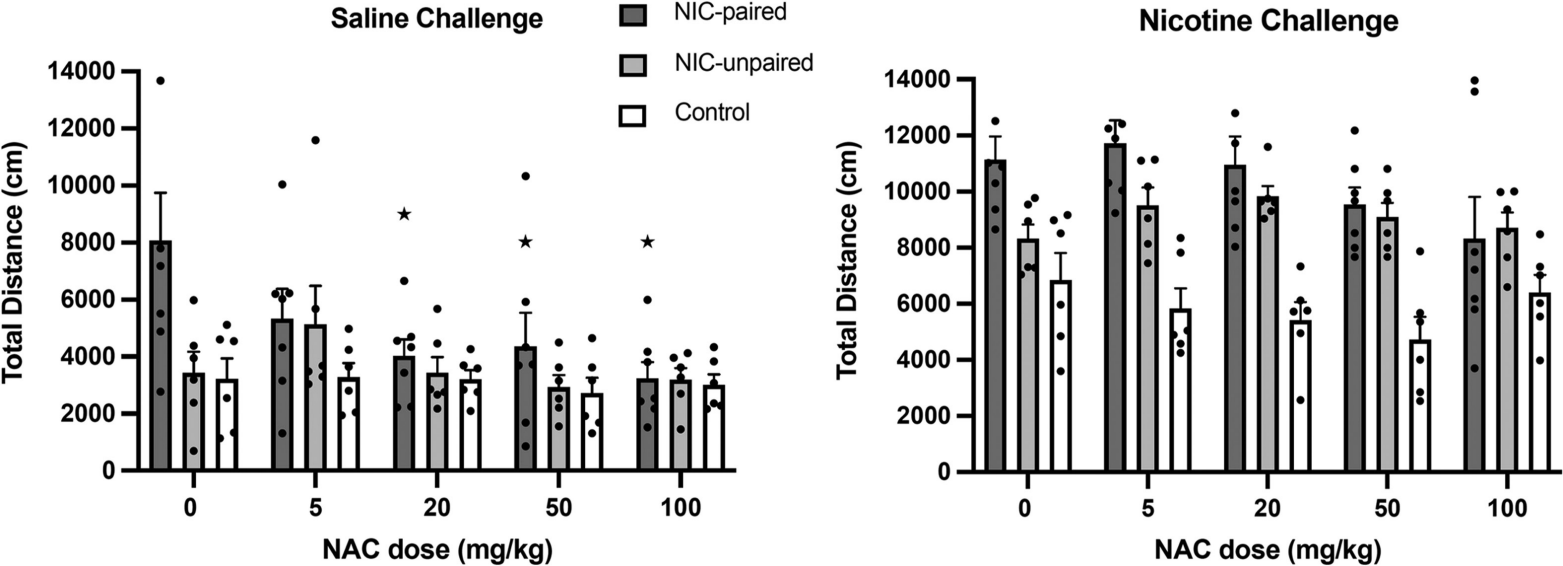
Total locomotor activity (distance travelled) (mean ± SEM) over the 45‐min test session after pre‐treatment with saline or NAC followed by saline or nicotine administration, for all three conditioned groups. *p < 0.05 significant difference in total distance (NIC‐paired group when pre‐treated with NAC and saline) compared to baseline (0 mg/kg NAC and saline) (one‐way ANOVA followed by LSD post hoc tests).

Figure 2 shows the effect of NAC on locomotor activity (total distance travelled) of nicotine‐ and saline‐treated rats over the 45‐min test session for all three groups (NIC‐paired, NIC‐unpaired and Control). In saline‐treated rats, there appeared to be a much higher level of activity exhibited by the nicotine‐paired groups compared to the nicotine‐unpaired and saline‐treated control groups (Figure 2). The significant nature of these interactions were analysed using multifactorial ANOVAs.

A three‐way ANOVA for repeated measures considered the interaction between the factors—conditioned group, NAC pre‐treatment (mg/kg) and nicotine administration which revealed a significant effect of NAC pre‐treatment [*F*(4,64) = 4.35, *p* = 0.004] and of nicotine administration [*F*(1,16) = 163.48, *p* = 0.0001] on total distance travelled, as well as an interaction between NAC*Group [*F*(8,128) = 2.58, *p* = 0.017] and Nicotine*Group [*F*(2,32) = 6.008, *p* = 0.011]. Within the NIC‐paired group, a significant effect of NAC pre‐treatment following saline administration was identified [*F*(4,24) = 3.71, *p* = 0.017], and pairwise comparisons revealed that NAC doses 20, 50 and 100 mg/kg significantly attenuated the total distance travelled, when compared to baseline levels of activity (0 mg/kg NAC) (*p* = 0.019, 0.014, 0.046) (Figure 2). A significant effect of NAC pre‐treatment was also observed following nicotine administration [*F*(4,24) = 3.464, *p* = 0.023]; however, subsequent pairwise comparisons did not reveal any significant differences at specific NAC doses. NAC pre‐treatment was shown to have no significant effect on the total distance travelled within the NIC‐unpaired or Control groups, when either saline or nicotine were administered before testing.

#### 3.1.2 First 20 min of Locomotor Activity

The locomotor activity (total distance travelled) in the first 20 min of the 45‐min test session was also analysed based on reports that nicotine plasma levels reach a maximum approximately 15–20 min following subcutaneous injection [53].

**FIGURE 3:**
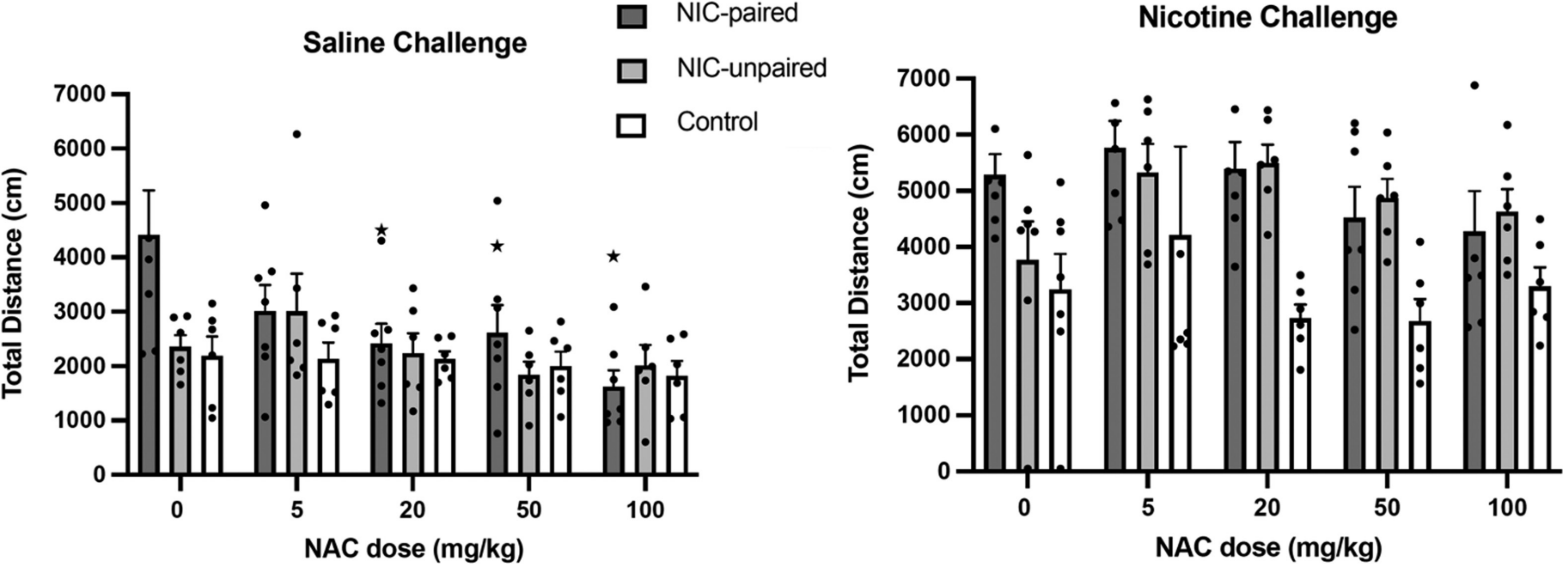
Total locomotor activity (distance travelled) (mean ± SEM) in the first 20 min of the test session after pre‐treatment with saline or NAC followed by saline or nicotine administration, for all three conditioned groups. *p < 0.05 significant difference in total distance (NIC‐paired group when pre‐treated with NAC and saline) compared to baseline (0 mg/kg NAC and saline) (one‐way ANOVA followed by LSD post hoc tests).

By examining the total distance travelled over the first 20 min of the test session, a significant three‐way interaction was found between NAC*Nicotine*Group factors [*F*(98,128)] = 2.14, *p* = 0.044]. Within the NIC‐paired group, a significant effect of NAC pre‐treatment following saline administration was established [*F*(4,24) = 4.872, *p* = 0.005] and pairwise comparisons showed that NAC doses 20, 50 and 100 mg/kg significantly reduced total distance travelled, with respect to baseline activity (*p* = 0.018, 0.023, 0.022) (Figure 3). A significant effect of NAC pre‐treatment was also identified following nicotine administration [*F*(4,24) = 3.02, *p* = 0.038]; however, pairwise comparisons did not display any significant differences at specific NAC doses. As with the 45‐min analysis, NAC pre‐treatment had no significant effect on the total distance travelled within the NIC‐unpaired or Control groups, when either saline or nicotine were administered before testing.

### 3.2 Nicotine Discrimination

Of the 12 rats trained to discriminate nicotine, one rat was discarded from the data analysis because it displayed no activity in all 18 test sessions. Therefore, a group size of 11 was used throughout for statistical analyses of the nicotine discrimination experiment.

**FIGURE 4:**
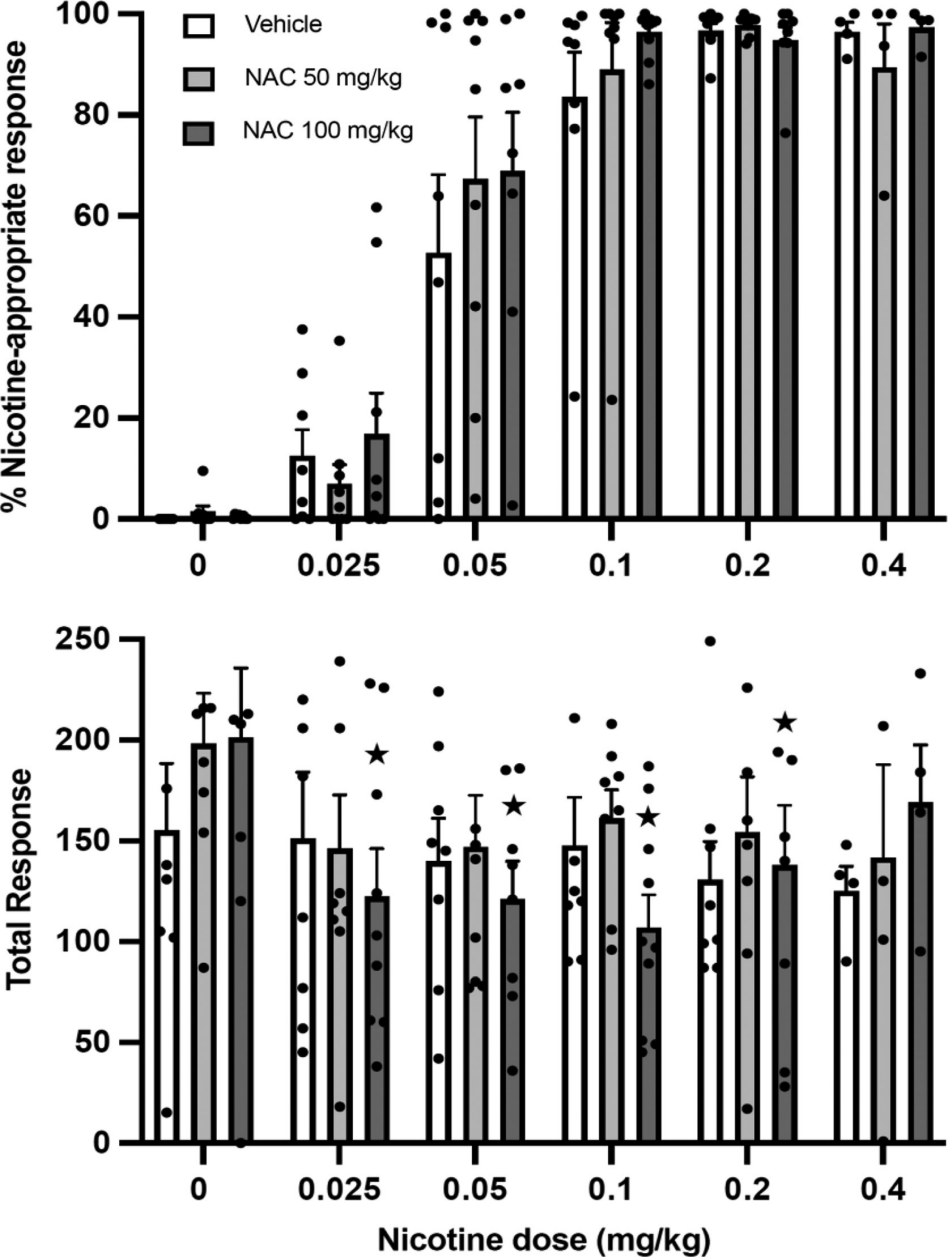
Operant lever press responses (mean ± SEM) made in the drug discrimination tests after pre‐treatment with vehicle or NAC followed by saline or nicotine administration (*n* = 11). *Top panel*: Percentage of responses on the nicotine‐paired lever. *Bottom panel*: Total number of responses on both levers. At 0.4 mg/kg nicotine dose, there was a low level of lever responding so data was only collected for *n* = 4 rats (for all NAC pre‐treatment doses), impacting the calculation of SEM. For statistical analysis, the mean of observed data was used in place of missing data. * *p <* 0.05 significant difference in total responses (100 mg/kg NAC pre‐treatment) at certain nicotine doses compared to baseline responding (0 mg/kg nicotine) (one‐way ANOVAs followed by LSD post hoc tests).

Figure 4 (top panel) shows the percentage of presses on the nicotine‐appropriate lever in response to a wide range of nicotine doses. Following treatment with vehicle, the dose response relationship was as expected; a maximal response on the nicotine‐appropriate lever at doses above 0.2 mg/kg nicotine (training dose) (0.2 mg/kg, 96.6 ± 1.5%; 0.4 mg/kg, 96.4 ± 2.0% [mean ± SEM]).

A two‐way ANOVA for repeated measures revealed significant effects of NAC pre‐treatment [*F*(2,20) = 4.24, *p* = 0.029] and nicotine test dose [*F*(4,40) = 115.40, *p* = 0.000] with regards to percentage nicotine‐appropriate responding; however, there was no significant interaction between the two factors, Nicotine*NAC. Despite this, post hoc analyses did not display any significant effects of NAC pre‐treatment, at any nicotine dose, on percentage nicotine‐appropriate responding. Therefore, NAC pre‐treatment had no significant effect on the dose response curve of nicotine discrimination.

Figure 4 (bottom panel) displays the total number of lever‐press responses, following each test. A two‐way ANOVA for repeated measures identified a significant effect of nicotine dose on the total number of responses [*F*(5,50) = 2.703, *p* = 0.031], with no overall effect of NAC pre‐treatment. A within‐group one‐way ANOVA for repeated measures (NAC 100 mg/kg) displayed a reduction in responding following NAC pre‐treatment at certain nicotine doses (0.025–0.2 mg/kg) compared to baseline responding at 0 mg/kg nicotine (*p* = 0.031, 0.029, 0.010, 0.047) (Figure 4).

## 4 Discussion

The present study demonstrates the specificity of N‐acetyl‐cysteine to attenuate the conditioned hyperactivity elicited by cues previously paired with nicotine administration. The levels of hyperactivity expressed by nicotine‐paired rats following saline injection were comparable to the levels observed by the nicotine‐unpaired groups of rats after administration of nicotine. The results from the locomotor study agreed with the hypothesis, showing NAC pre‐treatment to selectively attenuate the expression of the conditioned hyperactivity response, as the same dose range produced minimal effect on nicotine‐induced hyperactivity in the nicotine‐treated ‘unpaired’ group of rats and in the saline‐treated control group. In contrast, these doses of NAC did not modify the discriminative stimulus effects of nicotine, despite the highest doses of NAC supressing the total number of lever press responses.

The findings from this study support and challenge previous findings with NAC in preclinical models of nicotine dependence. The results from the locomotor activity experiment advance the findings of Ramirez‐Niño et al. [45], Moro et al. [42], Powell et al. [44], Goenaga et al. [41] and Nall et al. [43], which have all demonstrated that NAC can significantly attenuate cue‐induced reinstatement of nicotine‐seeking behaviour. Even though nicotine‐seeking behaviour was not specifically measured in our investigation, it demonstrated that NAC can similarly attenuate conditioned responses elicited by the presentation of stimuli previously paired with nicotine administration, as shown with discrete stimuli ([42]; Ramirez‐Niño et al. [45]. Furthermore, our results demonstrated an attenuation of the conditioned hyperactivity with doses of NAC greater than 20 mg/kg, which was significantly lower than those necessary to attenuate cue‐induced reinstatement of nicotine‐seeking behaviour (doses ≥ 60 mg/kg) [45]. Despite this, the lack of effect of NAC to modify the discriminative stimulus properties of nicotine, a measure of subjective effect, challenges a report from Ramirez‐Niño et al. [45] in which NAC attenuated nicotine self‐administration in rats. Rodent models of self‐administration and drug discrimination are routinely used to assess the abuse potential of psychoactive drugs [51], and therefore, are frequently employed when evaluating novel compounds for their ability to reduce abuse liability [54]. The various interoceptive effects of drugs denote the importance of utilising multiple preclinical models to assess prospective drug dependence treatments. However, given the different routes of nicotine administration and the different forms of motivation involved in these experiments, it will be difficult to compare the findings with NAC. Therefore, the present findings with NAC in the drug discrimination procedure should be considered with caution when drawing conclusions about the therapeutic potential of NAC to reduce nicotine dependence.

Doses of NAC greater than 20 mg/kg significantly attenuated the conditioned hyperactivity; a finding that was more apparent in the first 20 min of the test period compared to the total 45‐min test period. This may be due to the temporal onset of nicotine action on locomotor activity which was more likely to become associated with the locomotor test environment. A proposed explanation for the selective attenuation of the conditioned hyperactivity by NAC stems from evidence that implicates the glutamatergic system in the development and expression of behavioural sensitisation following chronic nicotine administration [48, 55]. Behavioural sensitisation to nicotine is thought to be mediated by both associative and non‐associative factors [56, 57]. Evidence from microdialysis studies suggest that elevated dopamine levels may be critical in the non‐associative forms of behavioural sensitisation to psychostimulants [58, 59]. In contrast, the glutamatergic system has been suggested, in part, to modulate the associative form of behavioural sensitisation [16]. The NAC receives glutamatergic afferents from many brain regions involved in conditioned stimuli [59, 60] and consequently, these stimuli are thought to enhance glutamate release in the nucleus accumbens, thus activating an increased number of NMDA receptors [16, 61]. Expression of behavioural sensitisation is believed to subsequently arise following increased dopaminergic transmission via the activation of NMDA receptors [16]. The present results demonstrating the selective action of NAC upon the conditioned hyperactivity support the hypothesis that acute NAC administration impacts the glutamatergic system that is crucial in the neuroadaptative process with repeated nicotine exposure. The inability of NAC to attenuate sensitised responses to nicotine suggests that the pharmacologically induced expression of behavioural sensitisation occurs via a different neural system.

Although the neurobiological mechanisms of NAC's action were not investigated in this study, it is widely accepted that NAC acts upon the glutamatergic system by activating system xc−, which subsequently increases the levels of extracellular glutamate and activating mGluR2/3 [24, 62]. Previous studies have shown that activation of mGlu2/3 receptors by the agonist LY379268 reduces responding to the presentation of nicotine‐associated cues and context [32, 63]. As a result, the ability of NAC to attenuate cue‐induced reinstatement of nicotine‐seeking behaviour has been suggested to be mediated via the activation of mGluR2/3s [42, 45] and is further complicated by the fact that NAC exhibits a sex‐specific efficacy for cue‐induced reinstatement of nicotine‐seeking behaviour [41].

NAC pre‐treatment did not modify the discriminative stimulus properties of nicotine, despite suppressing lever press responding during the test sessions. This suggests that the discriminative stimulus effect of nicotine has minimal involvement of glutamate in the mediation of these properties [64]. Although the full array of neurotransmitters underlying the discriminative stimulus properties remain unclear, the dopaminergic and cholinergic systems are suggested to be involved [65]. Zakharova et al. [59] evaluated the effects of the NMDA channel blockers MK‐801, dextromethorphan, and memantine, and the mGluR5 antagonist (MPEP) on the discriminative stimulus properties of nicotine which showed minimal interaction [64]. Our results corroborate these findings; given the action of NAC on the glutamatergic system and its lack of effect on the discriminative stimulus effects of nicotine, it is likely that nicotine's discriminative stimulus properties are mediated by other neural systems such as dopamine and serotonin [66, 67].

The implications of the present results lie primarily in the future development of NAC as a smoking cessation product. Our findings add to the current preclinical evidence of NAC's ability to attenuate behavioural measures of nicotine dependence, such as conditioned locomotor hyperactivity elicited by nicotine‐paired stimuli [42, 45, 46]. However, conditioned responses are scarcely displayed in tobacco smokers, and clinical trials evaluating NAC in smokers have yielded mixed outcomes, suggesting NAC may have limited efficacy [12, 28]. Although NAC administration has been shown to reduce the number of cigarettes smoked by participants in randomised clinical trials [28, 37, 38], one study indicated a trend towards decreased withdrawal symptoms in heavy smokers after 3.5 days of abstinence [68]. Another study reported that smokers rated the first cigarette after an abstinence period of 3.5 days as significantly less rewarding following NAC treatment compared to placebo, suggesting that the rewarding effects may have been attenuated [68].

Moreover, the reduction in the number of cigarettes smoked was only significant in the absence of alcohol, limiting NAC's potential therapeutic efficacy considering smokers are more vulnerable to alcohol use disorders than non‐smokers [69]. Two trials noted that NAC treatment suppressed cravings for cigarettes, which served to prolong abstinence [38, 70]. However, a recent meta‐analysis [39] demonstrated that although NAC suppresses cravings in patients with substance abuse, the evidence is weak. The analysis found no differences in the subgroup analysis according to the drug being abused (alcohol, cocaine, poly‐drugs, amphetamine and nicotine).

Despite the limited translation with most abused substances, there appears to be good concordance with NAC's preclinical profile to attenuate conditioned responses elicited by nicotine‐paired stimuli. Should this translate to humans, the data would predict better therapeutic potential for ‘tobacco chippers’ or intermittent smokers, populations that are more susceptible to smoking‐related cues, which play a primary role in smoking maintenance [71]. This contrasts with nicotine‐dependent smokers, where the importance of cues is equal to the pharmacological action of nicotine in the maintenance of dependence, suggesting that successful therapies ought to target both. Thus, the findings from this study propose that if NAC were to act in a similar selective manner in humans, its ability to specifically attenuate conditioned responses may limit its therapeutic potential for managing tobacco and nicotine dependence.

## Data Availability

Research data are not shared.
